# “*Primum, non nocere”*: The Epidemiology of Toxigenic *Clostridioides difficile* Strains in the Antibiotic Era—Insights from a Prospective Study at a Regional Infectious Diseases Hospital in Eastern Europe

**DOI:** 10.3390/antibiotics13050461

**Published:** 2024-05-17

**Authors:** Lidia Oana Stămăteanu, Claudia Elena Pleşca, Ionela Larisa Miftode, Aida Corina Bădescu, Doina Carmen Manciuc, Mihnea Eudoxiu Hurmuzache, Manuel Florin Roșu, Radu Ștefan Miftode, Maria Obreja, Egidia Gabriela Miftode

**Affiliations:** 1 Department of Infectious Diseases, “Grigore T. Popa” University of Medicine and Pharmacy, 700115 Iasi, Romania; lidia-oana.stamateanu@umfiasi.ro (L.O.S.); doina.manciuc@umfiasi.ro (D.C.M.); mihnea.hurmuzache@umfiasi.ro (M.E.H.); maria.n.obreja@d.umfiasi.ro (M.O.); egidia.miftode@umfiasi.ro (E.G.M.); 2 “St. Parascheva” Clinical Hospital of Infectious Diseases, 700116 Iasi, Romania; aida.badescu@umfiasi.ro (A.C.B.); florin.rosu@umfiasi.ro (M.F.R.); 3 Department of Preventive Medicine and Interdisciplinarity, “Grigore T. Popa” University of Medicine and Pharmacy, 700115 Iasi, Romania; 4 Surgical (Dentoalveolar and Maxillofacial Surgery) Department, “Grigore T. Popa” University of Medicine and Pharmacy, 700115 Iasi, Romania; 5 Department of Internal Medicine I (Cardiology), “Grigore T. Popa” University of Medicine and Pharmacy, 700115 Iasi, Romania; radu-stefan.miftode@umfiasi.ro

**Keywords:** *Clostridioides difficile*, presumptive 027/NAP1/BI, Romania, molecular diagnostic, antimicrobials, epidemiology

## Abstract

*Clostridioides difficile* infection (CDI), though identified nearly five decades ago, still remains a major challenge, being associated with significant mortality rates. The strains classified as hypervirulent, notably 027/NAP1/BI, have garnered substantial attention from researchers and clinicians due to their direct correlation with the severity of the disease. Our study aims to elucidate the significance of toxigenic *Clostridioides difficile* (CD) strains in the clinical and therapeutic aspects of managing patients diagnosed with CDI. We conducted a single-center prospective study, including patients with CDI from north-eastern Romania. We subsequently conducted molecular biology testing to ascertain the prevalence of the presumptive 027/NAP1/BI strain within aforementioned geographic region. The patients were systematically compared and assessed both clinically and biologically, employing standardized and comparative methodologies. The study enrolled fifty patients with CDI admitted between January 2020 and June 2020. Among the investigated patients, 43 (86%) exhibited infection with toxigenic CD strains positive for toxin B genes (*tcdB*), binary toxin genes (*cdtA* and *cdtB*), and deletion 117 in regulatory genes (*tcdC*), while the remaining 7 (14%) tested negative for binary toxin genes (*cdtA* and *cdtB*) and deletion 117 in *tcdC*. The presence of the presumptive 027/NAP1/BI strains was linked to a higher recurrence rate (35.56%, *p* = 0.025), cardiovascular comorbidities (65.1% vs. 14.2%, *p* = 0.016), and vancomycin treatment (55.8% vs. 14.3%, *p* = 0.049). The findings of our investigation revealed an elevated incidence of colitis attributed to presumptive 027/NAP1/BI. Despite the prevalence of the presumptive 027 strain and its associated heightened inflammation among the patients studied, no significant differences were observed regarding the clinical course or mortality outcomes.

## 1. Introduction

The Gram-positive anaerobic pathogen, *Clostridioides difficile* (CD), capable of spore formation within the gastrointestinal tract, continues to be the primary cause of healthcare-associated diarrhea. The existence of toxins associated with these bacteria renders the disease a substantial burden for both patients and global healthcare systems [1,2]. In 2019, CD was designated as a microorganism with an urgent threat level by the Centers for Disease Control and Prevention (CDC) [3].

Despite extended efforts in combating *Clostridioides difficile* infection (CDI) over the years, here, we are in the year 2024, and this infection continues to exert a significant impact on mortality rates, particularly among individuals aged above 65 years. Moreover, there is a noticeable escalation in recurrence rates and complications associated with this bacterium [4,5,6,7]. It is discernible that in recent years, the epidemiological landscape of CDI has experienced notable transformations, marked by a rise in incidence within populations lacking apparent risk factors. This includes individuals with no prior antibiotic exposure, young individuals, those without comorbidities, children, and pregnant women [8,9,10]. Conversely, therapeutic alternatives remain considerably constrained and have not demonstrated resounding success in effectively managing recurrent episodes [11,12,13,14].

Numerous risk factors contribute to the occurrence of CDI, with some of the most common ones being advanced age (≥65 years), antibiotic or proton pomp inhibitors usage, and prior hospitalization [15,16,17]. CDI exhibits a diverse array of symptoms, encompassing mild self-limited diarrhea, nausea, and abdominal pain, as well as severe and life-threatening complications, such as the development of toxic megacolon [18,19].

The challenge confronted by European health systems includes varied approaches to detection, surveillance, control, and prevention strategies across constituent states. For the diagnosis of CDI, various tests are available, including cytotoxicity assays, which, while considered the gold standard, are not recommended as routine assessments [20,21,22]. Instead, enzyme immunoassays (EIAs), nucleic acid amplification tests (NAATs), and tests detecting glutamate dehydrogenase (GDH) are commonly employed. Because the GDH test does not differentiate whether the strain is toxigenic or not, it should be complemented with a concurrent assay for toxin detection. To enhance diagnostic accuracy, algorithms combining two tests, such as GDH/NAAT and EIA, are frequently recommended [20,21,23].

The burdensome progression of the CDI epidemic is also attributed to the emergence of the hypervirulent epidemic strain: ribotype 027 (RT 027). Initially identified within healthcare facilities in Canada and the USA in 2003 [24], this strain presents unique challenges in terms of management, attributed to its potential for hypervirulence. This particular ribotype is linked to a more severe manifestation of the disease, with an observed increase in recurrence frequency, owing to its heightened resistance to antibiotic therapy [11,24,25,26,27,28]. RT 027 has the capability to generate the binary toxin CDT (CD transferase) which comprises CDTa, the enzyme component responsible for ADP ribosyltransferase activity, and CDTb, which facilitates toxin binding, entry, endosome formation, and subsequent delivery into the cytosol. The binary toxin released by this strain is linked to heightened secretion levels of toxins A and B, as well as a more severe manifestation of the disease [26,29,30].

Gaining a comprehensive understanding of the epidemiology of CDI is becoming progressively crucial for the implementation of effective preventive measures. In developing countries, there is no extensive nationwide surveillance program to analyze the circulating strains and ribotypes of CD. Consequently, the distribution of RT 027 remains inadequately understood. In our region, limited tests have been conducted to identify the RT 027, particularly in terms of its binary toxin secretion [31,32]. Thus, our objective is to investigate the presence of these data, along with the clinical and biological characteristics of patients harboring this strain. We aim to determine whether an Eastern European region will yield findings consistent with those published in other global regions.

## 2. Results

Between January and June 2020, fifty patients diagnosed with CDI through the immunochromatographic antigenic method for qualitative CD toxin detection underwent subsequent molecular biology testing. The objective was to determine the prevalence of the toxin-producing presumptive 027/NAP1/BI CD strains. Among the 50 patients analyzed, 43 exhibited infection with presumptive 027/NAP1/BI strains, positive for binary toxin genes (*cdtA* and *cdtB*) and deletion in position 117 in *tcdC*, while the remaining 7 tested negative for binary toxin and deletion 117.

**Figure 1:**
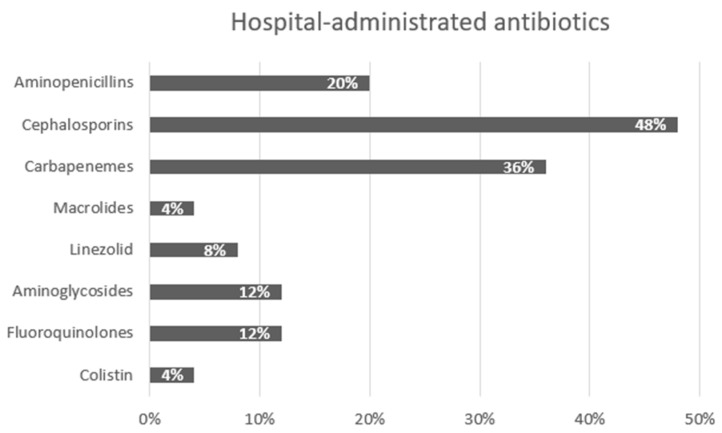
In-hospital-administered antibiotics for associated infections among the 50 enrolled patients.

Of the total 50 patients, 40% of the patients exhibited gastrointestinal symptoms prior to hospitalization, whereas 60% developed symptoms during their hospital stay. Predominant symptoms included watery diarrhea (94%) and semi-solid stools (76%), alongside abdominal discomfort (72%) and reduced appetite (60%). In total, 34% received antibiotic treatment prior to admission, with the most commonly administered being third-generation cephalosporins (35.2%), followed by fluoroquinolones (17.6%) and aminopenicillin/beta-lactamase inhibitors and carbapenems. The antibiotics administered to the patients during their hospitalization are graphically represented in Figure 1.

We conducted a comparative analysis of patient groups with CDI determined by presumptive 027/NAP1/BI strains and a group with CDI produced by negative strains for specific genes (*cdtA*, *cdtB*, and deletion 117 *tcdC*), examining clinical and paraclinical aspects through standardized and comparative methodologies. We designated the group of patients who tested positive for presumptive 027/NAP1/BI as R1, while the group who tested negative was labelled as R0.

The demographic parameters did not exhibit statistically significant differences between the two groups. However, from a percentage standpoint, the R1 group manifested a higher proportion of male patients (53.3% vs. 28.6%, *p* = 0.215). Additionally, patients from the R1 group had a greater mean age (66.51 years vs. 55.57 years, *p* = 0.127) and a longer hospitalization length (14.6 days vs. 10.14 days, *p* = 0.326) (Table 1).

While it was anticipated that significant differences would be evident in terms of signs, symptoms, and laboratory parameters between the two groups of patients, our observations indicate otherwise, with no significant differences identified (Table 2 and Table 3). Nevertheless, it is noteworthy to mention that the inflammatory syndrome, evaluated through C-reactive protein values, erythrocyte sedimentation rates, and fibrinogen, exhibited a more pronounced escalation in patients from group R1 in comparison to those in group R0.

In terms of comorbidities linked to the toxin-producing presumptive 027/NAP1/BI CD strains, our statistical analysis revealed a higher frequency of association with cardiovascular comorbidities (65.1% vs. only 14.2%, *p* = 0.016). The prevalence of other comorbidities remained comparable between the two groups (Table 4).

Antibiotic treatment was administered prior to hospitalization in 39.4% of patients in the R1 group and 28.6% in the R0 group. Regarding the occurrence of CDI during hospitalization, our observations revealed that 53.5% of patients in group R1 received treatment before CDI, while a comparable situation was noted in 28.6% of patients in group R0. Conversely, statistical analysis did not affirm the significance of comparing the antibiotic groups administered to patients in the two established groups (Table 5).

For the antibiotic management of CDI, a statistically significant difference was observed, indicating a more frequent use of vancomycin in patients with presumptive 027/NAP1/BI-positive strains (55.8%, *p* = 0.049), while those who tested negative exhibited a higher frequency of treatment with metronidazole (71.4%, *p* = 0.027). Fecal microbiota transplantation was performed in only two cases among patients in group R1 (4.7%) (Table 6).

In our statistical analysis of the outcomes of CDI, the presence of the epidemic presumptive 027/NAP1/BI strain was linked to a higher recurrence rate (35.56%, *p* = 0.025) (Table 7). In terms of mortality, group R1 experienced six fatalities, contrasting with group R0, where the count registered a single fatality. Another noteworthy observation in our statistical analysis was that all deceased cases were male, whereas the survivors were predominantly female (58.1% vs. 41.8%, *p* = 0.001).

We aimed to explore the parameters linked to the risk of unfavorable progression among patients with toxigenic CD strains. To achieve this, we conducted logistic regression analysis to ascertain the potential associations between disease prognosis and certain parameters (such as calprotectin above 200 μg/g, the presence of toxin B by PCR (polymerase chain reaction), and the number of stools). Consequently, in the initial model featuring the independent variable of calprotectin above 200 μg/g, we observed a significant association with an unfavorable outcome (OR 1.29; 95% CI 0.076–3.979; *p* = 0.046), indicating a 1.29-fold higher risk of mortality. Following, in the subsequent model incorporating calprotectin above 200 μg/g alongside the presence of toxin B detected by PCR (OR 2.15; 95% CI 0.610–12.543; *p* = 0.001), a two-fold elevated mortality risk was identified. In the last model, we also included stool frequency (OR 0.82; 95% CI 0.680–0.996; *p* = 0.046) as an independent variable in addition to the aforementioned two factors (calprotectin above 200 μg/g—*p* = 0.029 and the presence of toxin B detected by PCR—*p* = 0.012), once again highlighting an increased risk of a poor outcome. The quality of these models basically resides in the synergistic prognostic value between a specific calprotectin cut-off value (200 μg/g) and by a comprehensive clinical (number of stools) and microbiological (toxin B) assessment (Table 8).

**Figure 2:**
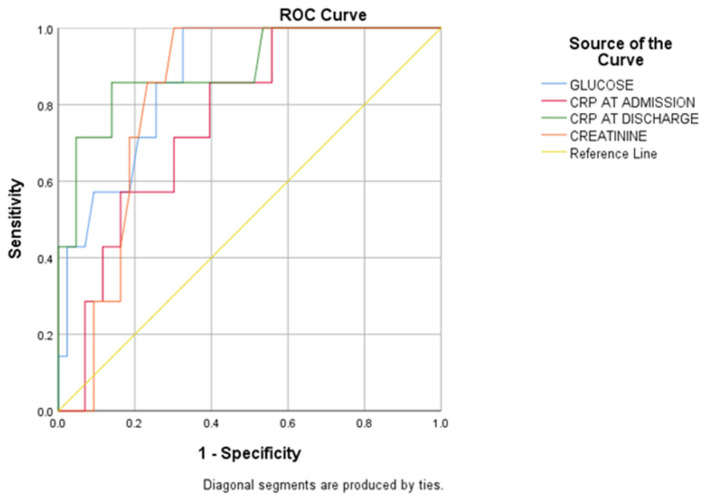
ROC curves for laboratory parameters associated with mortality among group R1.

We aimed to identify predictors for an increased mortality risk. Upon preliminary analysis, it was noted that the presumptive 027/NAP1/BI CD strain exhibited no significant correlation with inflammatory markers upon admission or discharge, or with serum biomarker levels indicative of liver or kidney function, mortality rates, or the recurrence of CDI. By performing ROC analysis (Table 9), we observed that blood glucose, serum creatinine, and C-reactive proteins (assessed at admission and at discharge) exhibited AUC > 0.750, thus highlighting the predictive role of these modifiable variables in the management of patients with CDI (Figure 2).

Subsequently, considering that all these predictors associated with a poor prognosis can coexist in varying proportions in the same patient, through a multiple regression model, we aimed to create a more accurate mortality prediction model. Despite the fact that increased fasting blood glucose (Model 3) was per se a significant mortality predictor (R = 0.499 and R^2^ = 0.233), superior to the model comprising toxin B and calprotectin 200 (R = 0.201 and R^2^ = 0.040), we noticed that a composed model based on routinely assessed laboratory parameters (Model 5) was highly predictive of an increased mortality rate (R = 0.566 and R^2^ = 0.321) (Table 10). Basically, more than 30% of the mortality rate could be related to the variation in ALT, platelet count, CRP at admission, creatinine, glucose, and serum urea. These models aim to be easy-to-use and reproducible tools for initial prognosis assessment in various clinical settings, without requiring advanced microbiological assays. Moreover, the Durbin–Watson value of 2.21 for this model (within the normal range of 1.5–2.5) expresses a low chance of autocorrelation, thus emerging as the most appropriate of all the designed models.

## 3. Discussion

Understanding the epidemiology of CDI is of growing significance, given the global impact of this disease. In terms of the prevalence of CDI in the United States of America (USA), a meta-analysis conducted in 2020 estimates an incidence of 8.3 cases per 10,000 patient days [33]. Additionally, according to the most recent surveillance report provided by the CDC, the overall crude incidence rate stands at 110.2 cases per 100,000 people [33]. In Europe, the average incidence of this disease, as reported in the most recent European Centre for Disease Prevention and Control (ECDC) report, was 3.48 cases per 10,000 patient days [34].

The surge in the global incidence of CDI cases can be attributed to a confluence of epidemiological and genetic determinants, notably the widespread dissemination of hypervirulent clones, such as 027/NAP1/B1, which are associated with high mortality rates, particularly among the elderly [35,36,37]. This phenomenon is compounded by inadequate prevention measures and a dearth of effective surveillance protocols targeting cases with heightened transmission potential [38,39]. Additionally, uncertainty persists regarding optimal timing for patient testing [40,41].

In Romania, the most recent report from 2020 indicates a nearly 50% reduction in the incidence of CDI compared to the preceding year. The primary diagnostic method employed in this report involved the antigenic detection of toxins A and B, which constituted 99.3% of the tests conducted, while the remaining 0.7% utilized PCR [42].

Although recognized for its significant role in antibiotic-associated diarrhea within both nosocomial and community settings in the US and Europe, there is a paucity of studies detailing the molecular epidemiological landscape of CDI in Romania, particularly in the north-eastern region [31,32,43,44]. Presumably, this can be attributed to the elevated cost associated with the ribotyping test, rendering it financially inaccessible for many hospitals in Romania.

In 2005 and 2006, the Netherlands documented several epidemic surges in CDI, characterized by the prevalence of RT 027 and toxinotype III. These outbreaks posed significant challenges in terms of epidemiological management and control [45]. Severe cases of CDI, caused by hypervirulent strains, have likewise been documented in the United States, with a 30-day mortality rate exceeding 13% in contrast to other strains associated with a lower 30-day mortality rate [37,46,47]. As is well recognized, exposure to antibiotics constitutes the primary risk factor for CDI. Notably, fluoroquinolones, cephalosporins, clindamycin, and aminopenicillins are associated with the highest risk of inducing CDI [48,49,50]. A study indicated that all fluoroquinolones possess the potential to trigger CDI, including the strain RT 027, despite their anaerobic activity. Resistance was observed in the case of moxifloxacin [51].

In Romania, epidemiological dynamics exhibit a continuous trend, characterized by the escalating incidence of community-acquired CDI. Understanding the factors contributing to CDI occurrence within communities and social care settings is crucial for implementing effective prevention strategies [52,53]. Moreover, the most important strategy in reducing the incidence of this pathology would be a dynamic assessment of the antibiotic susceptibility rates of the most common pathogens in order to limit exposure to multiple antibiotics; this aspect is particularly important in Romania, where both antibiotics consumption per capita and antimicrobial resistance rates are among the highest from Europe [54,55,56,57,58].

Recent national studies conducted in the western and southern regions revealed that a significant portion of CDI cases, ranging from 68% to 82.6%, were attributed to RT 027. Alarmingly, around 40% of these cases required hospitalization, posing a risk for further outbreaks in healthcare facilities [31,32,59].

Our study revealed the high prevalence of CDI patients infected with the toxin-producing presumptive 027/NAP1/BI CD strain (86% vs. 14%). The male-to-female ratio was similar across both groups. Patients infected with the toxigenic CD strain (presumptive 027/NAP1/BI) had a higher mean age compared to those who tested negative for these particular strains (66.51 vs. 55.57 years). Despite a wide age range (24 to 93 years), the predominance of elderly individuals underscores the heightened susceptibility of this population demographic to CDI, irrespective of the strains involved.

Another finding outlined in a national study from the Romania northwest region highlights the different involvement of the 027/NAP1/BI strain in the etiopathogenesis of CDI among community-acquired cases versus those occurring in hospitalized patients (53.5% vs. 82.6%). Moreover, the same study highlighted a link between the hospitalization length and the type of infection, with significantly shorter hospital stay among patients with community-acquired CDI [59]. A 2018 study conducted in the USA underscored a substantial proportion of CDI cases associated with healthcare settings attributed to the 027/NAP1/BI strain [60]. Likewise, a study by Turner et al. published in 2019 identifies parallels to the findings elucidated in our investigation [61].

In our study, the patients under investigation remained hospitalized throughout the entire course of the disease, precluding any comparison with cases originating from the community. Among these patients, those in group R1 exhibited a slightly prolonged mean hospitalization length (14 days compared to 10 days), likely reflective of the severity of their condition. Intriguingly, individuals in group R1 experienced delays in hospital admission and subsequent diagnosis, with some being admitted as late as the 30rd day of illness progression. This delay may be attributed to potential misdiagnoses stemming from the absence of specific testing for CD and empirical efforts to manage gastrointestinal symptoms.

CD is a common cause of persistent diarrhea, particularly affecting elderly individuals, with approximately 80% of cases occurring in patients over 65 years old. However, the exact reasons for this susceptibility are not fully understood. One possible explanation involves a decline in the resilience of natural barriers to such infections [62,63,64]. Susceptibility to CDI is associated with disturbances in the balance of the intestinal microbiota. Despite its heightened virulence, the 027/NAP1/BI strain of CD demonstrates limited competitiveness with indigenous microflora within the gut. Consequently, in a normobiotic colon, the probability of disease manifestation is noticeably diminished [65,66].

Warny et al. demonstrated that the NAP1/B1/027 strain exhibits the hyperproduction of toxins, with enterotoxin A levels elevated by 16-fold and toxin B levels by 23-fold compared to control strains [67].

It is well established that individuals with comorbidities exhibit increased susceptibility to various pathogens. In addition to the classic risk factors for CDI, such as diabetes myelitis, gastrointestinal disorders, and renal insufficiency, the literature also identifies chronic cardiovascular diseases as potential contributors [68,69,70,71]. Similarly, the statistically significant difference observed in our study pertained to the association of chronic cardiovascular comorbidities in group R1 (65.1% vs. 14.2%, *p* = 0.016).

Mortalities attributable to infection with this specific strain of CD have yielded conflicting findings in research studies. A retrospective analysis led by Bauer et al. suggested that the NAP1/B1/027 strain does not correlate with severe manifestations of the disease, nor does it exhibit heightened in-hospital mortality or increased recurrence rates [72]. Sirad et al. arrived at a similar conclusion in their research, noting that despite the heightened toxin production associated with NAP1/B1/027 compared to other strains, the lower spore count was linked to less severe presentations of the disease [73]. These findings are corroborated by additional cohort and case–control studies [74,75]. Conversely, Rao et al. elucidated through a cohort study that the 027 RT is notably associated with a higher incidence of severe forms of CDI (OR 1.73; 95% CI 1.03–2.89; *p* = 0.037) and elevated mortality rates (OR 2.02; 95% CI 1.19–3.43; *p* = 0.009) compared to other ribotypes [36,76]. Another study conducted in Quebec revealed that this specific strain produces twice as many severe forms of CDI compared to other strains examined [77]. Several explanations could account for the disparities among the findings of studies on this subject, including variations in study design, patient selection criteria, sample sizes, and laboratory techniques employed for detecting specific toxins.

In our investigation, among the 43 patients in group R1, 6 (13.9%) experienced fatal outcomes, while only 1 (14.2%) of the 7 in group R0 succumbed to this adverse outcome. From a numerical standpoint, there were no notable differences between the two groups. Additionally, the recurrence rate was notably elevated among individuals in group R1 (32.56%; *p* = 0.025). Contrarywise, none of the patients in group R0 exhibited recurrences.

Considering the conflicting findings present in the literature, selecting the most suitable approach for treating CDI should consider various factors. These include the specific characteristics of the affected individual; the severity of the inflammatory and diarrheal syndromes; the degree of dehydration; renal function, as indicated by glomerular filtration rate; serum albumin levels; pre-existing medical conditions; and, importantly, the immunological status [78].

The keystone of the pathophysiological mechanisms underlying CDI lies in the production of two distinct toxins, A and B. Toxin A acts as an enterotoxin, while toxin B functions as a cytotoxin, both possessing a substantial molecular weight and exhibiting specific binding affinity to receptors on the colonic mucosa. These toxins elicit significant inflammatory reactions, being released during the late logarithmic growth phase and the stationary phase [79,80]. While the enterotoxin primarily affects the actin within target cells, resulting in their destruction and the consequent necrosis of epithelial cells, cytotoxin B induces intracellular junctional disruptions, leading to heightened vascular permeability and the onset of hemorrhagic events [81,82].

In our study, both toxin B and binary toxin were detected in all patients from group R1 (100%). This indicates the heightened incidence among patients with CDI caused by presumptive 027/NAP1/BI epidemic strains, which is accountable for an accelerated toxin production process.

A study conducted among individuals involved in an outbreak attributed to CD identified that 52% of participants were asymptomatic carriers of toxigenic strains. Notably, at least one-third of these carriers belonged to the NAP1/027 type [83]. These findings underscore the potential role of asymptomatic carriers in the transmission of virulent strains of CD [40,84].

Fecal calprotectin (FC) stands out as a biomarker of notable significance in predicting severe forms of CD colitis. Elevated levels of calprotectin in fecal samples are strongly associated with heightened inflammation of the colonic mucosa, thereby reflecting the severity of the disease [85,86,87]. One study revealed elevated FC levels in patients with RT 027 compared to those without this ribotype (317 vs. 60 μg/g; *p* = 0.0014) [35]. Given these considerations, we opted to analyze FC levels in our patient cohort. Subsequently, our investigation unveiled markedly elevated FC levels (>200 μg/g) in 32.5% of individuals afflicted to the group R1, contrasting with only one case of elevated FC observed among those in group R0.

The primary risk factor associated with the onset of CDI is antibiotic therapy, with the type and duration of antibiotic treatment being particularly significant. The global rise in severe infections, coupled with the proliferation of microbial resistance, has necessitated the widespread and prolonged utilization of broad-spectrum antibiotics, resulting in significant dysbiosis [88,89,90]. Essentially, the disruption of the gut microbiota caused by medications forms the basis of CD colitis, which tends to complicate the progression of pre-existing infectious and non-infectious conditions. The escalating use of antibiotics, both within the community and hospital settings, is a well-established reality on a national scale [91,92].

Among patients in group R1, 39.4% received antibiotic therapy in the four weeks preceding admission to our clinic. Moreover, 53.5% of group R1 required antibiotic therapy during hospitalization, while this figure was 28.6% for group R0. The predominant antibiotics administered to the first group of patients included cephalosporins (25.6%), carbapenems (20.9%), aminopenicillins (11.6%), aminoglycosides (9.3%), and fluoroquinolones (7%). In contrast, the second group of patients received cephalosporins, macrolides, and carbapenems equally, each constituting 14.3% of the antibiotics administered. While the discontinuation of antibiotic therapy is the primary therapeutic recommendation for CDI, there are situations where this may not be feasible due to concurrent infections in patients. This consideration applies to the subjects of our study, particularly those who developed colitis caused by toxigenic CD strains.

In terms of antibiotic resistance among NAP1/B1/027 strains, a study revealed reduced sensitivity to clindamycin, levofloxacin, moxifloxacin, ciprofloxacin, and rifampicin, while remaining adequately sensitive to metronidazole and vancomycin [93]. In a comparative investigation, the antibiotic resistance profile of the NAP1/B1/027 strain was contrasted with the RT 027 negative group, revealing significant differences. The NAP1/B1/027 strain exhibited a pronounced resistance to moxifloxacin, with a prevalence of 92.2% compared to a mere 11.2% in the NAP1/B1/027-negative group. Similarly, notable resistance rates were observed in the case of ceftriaxone, with 78.2% resistance in the first group versus 15.7% in the second group. In the same investigation, RT 027 exhibited substantially lower sensitivity to metronidazole, with a fourfold higher minimum inhibitory concentration compared to other ribotypes (4 µg/mL vs. 1 µg/mL). Furthermore, RT 027 demonstrated a twofold reduction in sensitivity to fidaxomicin (2 µg/mL vs. 1 µg/mL), while maintaining similar sensitivity to vancomycin, as observed in other ribotypes [94]. These findings support the notion of a diminished therapeutic efficacy of metronidazole and fidaxomicin in patients infected with RT 027 compared to those infected with other ribotypes, as well as an elevated risk of relapse among the former. Nevertheless, the CDI Diagnostic and Treatment Guidelines remain applicable regardless of the strain type, including NAP1/B1/027 strains. Although research on this matter has not consistently revealed the resistance of NAP1/B1/027-positive strains to fidaxomicin, isolated cases have been reported where, due to adverse outcomes after fidaxomicin treatment, and even subsequent to fecal microbiota transplant, therapy involving intravenous immunoglobulins was opted for [95].

The etiological treatment administered to the patients in our study revealed significant differences. Specifically, in the R1 group, the prevalence of vancomycin treatment was notable (55.8%, *p* = 0.049), whereas in the R0 group, a similar pattern was observed with metronidazole treatment (71.4%, *p* = 0.027). The etiological treatment regimen extended over a period of 10–14 days, resulting in favorable clinical outcomes. None of the patients were prescribed fidaxomicin, and only 6.7% of those in the R1 group underwent fecal microbiota transplant.

In previous studies, advanced age, heightened comorbidity, renal impairment, and cancer emerged as predictors of mortality in CDI [96,97,98,99]. Moreover, the presence of RT 027 was frequently identified as a prognostic indicator for mortality [35,76]. In our study, regarding predictors of mortality in patients with presumptive 027/NAP1/BI-positive CD strains, a model selection approach was employed to enumerate all potential models derived from the established predictors. This method highlighted that patients with elevated FC levels (OR 1.29; 95% CI 0.076–3.979; *p* = 0.046), the presence of toxin B (OR 2.15; 95% CI 0.610–12.543; *p* = 0.001), and increased stool frequency (OR 0.82; 95% CI 0.680–0.996; *p* = 0.046) are at a higher risk of mortality. Another simple, easy-to-use, and reproducible predictive model was based on the routinely assessed laboratory parameters, such as platelet count, CRP, fasting glucose, and liver and kidney function.

## 4. Materials and Methods

The research was structured as a prospective study conducted at the “St. Parascheva” Clinical Hospital of Infectious Diseases, a tertiary-care facility catering to patients with infectious diseases from the entire northeast region of the country. Commencing in January 2020, the study concluded in June 2020.

The inclusion criteria were adult age (>18 years) and a positive antigenic test for CD toxins A and B (by chromatographic immunoassay qualitative tests). We included patients with CDI who presented digestive symptoms before admission and patients who developed CDI during hospitalization. Patients were excluded if they were under 18 years of age.

Stool samples were obtained from 50 hospitalized patients. Each patient was provided with a sterile container and instructed to collect a stool sample. Upon collection, all stool samples underwent testing for CD GDH, toxin A, and toxin B using immunoassay techniques (CerTest Biotec S.L., Zaragoza, Spain). Each collected stool sample was prepared for antigenic extraction and dispensed into three separate circular windows (GDH, toxin A, and toxin B), following the manufacturer’s guidelines. Results were recorded after 10 min. A negative result was indicated by a green line (control line) appearing in all three circular windows (GDH, toxin A, and toxin B), while a positive result was identified by the presence of a red line in any of these circular windows (GDH, toxin A, and toxin B).

All stool specimens identified as positive through antigenic immunoassay testing were subjected to the molecular detection of CD using the Xpert *C. difficile* BT (Cepheid, Sunnyvale, CA, USA), a qualitative in vitro diagnostic test, performed on the GeneXpert^®^ System device. The Xpert CD BT assay was conducted in accordance with the manufacturer’s protocols. This test is a multiplex PCR used to detect the toxin B coding gene (*tcdB*), binary toxin genes (*cdtA* and *cdtB*), and single-nucleotide deletion at position 117 in the *tcdC* gene in CD strains. The test has the capacity to detect toxigenic CD strains and presumptively differentiate NAP1/B1/027 strains (positive for 117 deletion in *tcdC* genes) directly from stool samples, offering an impressively brief response time of just 45 min. There was no post-discharge follow-up regarding the readmission of the patients.

Patient data, e.g., on clinical and demographic profiles, laboratory parameters, comorbidities (e.g., cardiovascular diseases and diabetes mellitus), prescribed antibiotic regimens, and subsequent clinical responses, were systematically extracted from medical records.

The dataset was compiled and analyzed using SPSS (IBM Corp. Released 2021. IBM SPSS Statistics for Windows, Version 28.0. Armonk, NY, USA: IBM Corp). Multivariate logistic regression was conducted to ascertain risk factors for mortality and composite severe outcomes linked to RT 027. Group disparities were evaluated via independent t-tests or one-way ANOVA, as applicable. Odds ratios (ORs), 95% confidence intervals (CIs), and *p*-values were computed, with values below 0.05 considered statistically significant. Variables with *p* < 0.3 on univariate analysis were incorporated into a logistic regression model to identify independent risk factors associated with presumptive 027/NAP1/BI.

## 5. Conclusions

In our study, we observed the heightened prevalence of toxigenic presumptive 027/NAP1/BI strains in our geographical area (86%), which exhibited a statistically significant association with an elevated risk of disease recurrence, as well as concurrent cardiovascular comorbidities. Concerning the management of CDI, patients infected with presumptive 027 strains demonstrated statistical significance in their response to vancomycin treatment. The predictors of mortality among patients with toxigenic-producing presumptive 027/NAP1/BI-positive strains included elevated fecal calprotectin levels, increased stool frequency, and the presence of toxin B. Furthermore, despite the epidemic strain being frequently linked to heightened toxin B and binary toxin production, no notable disparities were observed regarding the disease presentations in patients infected with presumptive 027-positive strains versus those afflicted with different strains. Given these considerations, and noting the absence of guidelines recommending specific antibiotic therapies based on ribotype, it is imperative that the management of CD colitis cases is guided by careful clinical assessment. This should involve evaluating the severity of symptoms, the presence of comorbidities, and relevant laboratory findings to determine the most appropriate course of action.

## 6. Study Limitations

It is essential to acknowledge the limitations of our study, including the small number of included patients and the single-center design. Furthermore, our study is constrained by the inability of the test to use Xpert *C. difficile* BT to specifically identify 027/NAP1/BI, potentially leading to the detection of other ribotypes, aside from 027/NAP1/BI [100]. Consequently, our reported results are presented as *presumptive*, given this inherent limitation.

## Figures and Tables

**Table 1:** Demographic characteristics of patients.

Demographic Parameters	Toxin-Producing CD Strains (Presumptive 027/NAP1/BI)		*p*-Value
	Positive R1 (n = 43)	Negative R0 (n = 7)	
Sex, n (%)			0.215
Male	23 (53.5%)	2 (28.6%)	
Female	20 (46.5%)	5 (71.4%)	
Age, years	66.51 ± 16.06	55.57 ± 24.11	0.127
mean ± SD, limits	(24–81)	(30–93)	
Environment, n (%)			0.857
Urban	23 (53.5%)	4 (57.1%)	
Rural	20 (46.5%)	3 (42.9%)	
Hospitalization length, mean ± SD, limits	14.60 ± 11.58 (1–63)	10.14 ± 5.79 (3–19)	0.326
Sick day at admission, mean ± SD, limits	5.58 ± 6.25 (1–30)	2.14 ± 1.07 (1–4)	0.156

Abbreviations: SD—standard deviation.

**Table 2:** Clinical characteristics of patients.

Signs and Symptoms	Toxin-Producing CD Strains (Presumptive 027/NAP1/BI)		*p*-Value
	Positive R1 (n = 43)	Negative R0 (n = 7)	
Diarrhea before admission, n (%)	33 (76.7%)	5 (71.4%)	0.764
Diarrhea after admission, n (%)	43 (100%)	7 (100%)	1.000
No. of stools on admission, mean ± SD, limits	5.86 ± 3.04	6.00 ± 4.08	0.915
Watery stools, n (%)	41 (95.3%)	6 (85.7%)	0.378
Semiconsistent stools, n (%)	33 (76.7%)	5 (71.4%)	0.764
Abdominal pain, n (%)	31 (72.1%)	5 (71.4%)	0.971
Vomiting, n (%)	15 (34.9%)	3 (42.9%)	0.687
Loss of appetite, n (%)	25 (58.1%)	5 (71.4%)	0.498
Fever, n (%)	9 (20.9%)	1 (14.3%)	0.673
Chills, n (%)	4 (9.3%)	0 (0.0%)	0.261
Astheny, n (%)	12 (27.9%)	1 (14.3%)	0.422
Headache, n (%)	3 (7.0%)	2 (28.6%)	0.124

Abbreviations: SD—standard deviation.

**Table 3:** Results of laboratory characteristics analysis for the two groups studied.

Parameters	Toxin-Producing CD Strains (Presumptive 027/NAP1/BI)		*p*-Value
	Positive R1 (n = 43)	Negative R0 (n = 7)	
WBC (cells/μL), mean ± SD, limits	12,464 ± 6901 1430–27,190	12,872 ± 8394 3990–29,170	0.888
Neutrophils (%), mean ± SD, limits	93.94 ± 92.92 50.3–633	82.11 ± 7.75 68.5–92.3	0.743
Lymphocytes (%), mean ± SD, limits	18.51 ± 14.90 2.0–72.2	11.73 ± 5.77 6.1–22.9	0.244
Thrombocytes (cells/μL), mean ± SD, limits	256.90 ± 95.45 7–1196	205.29 ± 95.45 43–283	0.484
CRP at admission (mg/dL), mean ± SD, limits	98.20 ± 90.91 0.49–440	88.13 ± 100.01 2.50–252	0.790
ESR (mm/hr), mean ± SD, limits	62.44 ± 40.55 0–140	36.57 ± 19.21 26–80	0.106
Fibrinogen (mg/dL), mean ± SD, limits	3.58 ± 1.60 1.23–9.35	3.16 ± 1.07 1.26–4.26	0.510
Total proteins at admission, mean ± SD, limits	60.10 ± 20.03 0–92.13	72.16 ± 7.24 60.93–81.50	0.125
INR, mean ± SD, limits	0.78 ± 0.76 0–2.85	0.90 ± 1.05 0–2.86	0.717
Sodium (mmol/L), mean ± SD, limits	142.44 ± 2.96 132–148	140.80 ± 2.92 137–145	0.180
Potassium (mmol/L), mean ± SD, limits	3.81 ± 0.52 2.56–5.07	3.84 ± 0.54 3.01–4.63	0.872
Chloride (mmol/L), mean ± SD, limits	101.17 ± 3.03 94–107.30	99.10 ± 4.36 90.60–104.20	0.122
Glucose (mg/dL), mean ± SD, limits	121.84 ± 69.80 61–512	101.14 ± 16.16 82–124	0.442
Urea (mg/dL), mean ± SD, limits	54.47 ± 37.92 9–164	30.86 ± 14.19 17–55	0.112
Creatinine (mg/dL), mean ± SD, limits	1.36 ± 1.10 0.63–7.00	0.94 ± 0.20 0.65–1.25	0.324
ALT (U/L), mean ± SD, limits	42.44 ± 45.79 6–249	21.00 ± 7.35 12–32	0.226

Abbreviations: ALT—alanine aminotransferase; CRP—C-reactive protein; ESR—erythrocyte sedimentation rate; INR—international normalized ratio; LDH—lactate dehydrogenase; SD—standard deviation; WBC—white blood cell count.

**Table 4:** Summary of comorbidity analysis for the two groups studied.

Comorbidities	Toxin-Producing CD Strains (Presumptive 027/NAP1/BI)		*p*-Value
	Positive R1 (n = 43)	Negative R0 (n = 7)	
Cardiovascular, n (%)	28 (65.1%)	1 (14.2%)	**0.016**
Diabetes, n (%)	8 (18.6%)	0 (0.0%)	0.104
Gastroenterological, n (%)	21 (48.8%)	3 (42.9%)	0.769
Pulmonary, n (%)	3 (7.0%)	1 (14.3%)	0.541
Obesity, n (%)	4 (9.3%)	0 (0.0%)	0.261
Neurological, n (%)	8 (18.6%)	0 (0.0%)	0.104
Rheumatological, n (%)	3 (7.0%)	0 (0.0%)	0.333
Psychiatric, n (%)	7 (16.3%)	0 (0.0%)	0.130
Endocrinological, n (%)	7 (16.3%)	0 (0.0%)	0.130
Chronic kidney disease, n (%)	9 (20.9%)	0 (0.0%)	0.082
Oncological, n (%)	7 (16.3%)	2 (28.6%)	0.456
Dialysis, n (%)	2 (4.7%)	0 (0.0%)	0.432

**Table 5:** Overview of antibiotics administered to patients prior to the onset of CDI.

Antibiotics	Toxin-Producing CD Strains (Presumptive 027/NAP1/BI)		*p-*Value
	Positive R1 (n = 43)	Negative R0 (n = 7)	
Cephalosporins, n (%)	11 (25.6%)	1 (14.3%)	0.496
Aminopenicillins, n (%)	5 (11.6%)	0 (0.0%)	0.206
Macrolides, n (%)	0 (0.0%)	1 (14.3%)	0.140
Carbapenems, n (%)	9 (20.9%)	1 (14.3%)	0.673
Linezolid, n (%)	2 (4.7%)	0 (0.0%)	0.432
Fluoroquinolones, n (%)	3 (7.0%)	0 (0.0%)	0.333
Aminoglycosides, n (%)	4 (9.3%)	0 (0.0%)	0.261
Colistin, n (%)	1 (2.3%)	0 (0.0%)	0.581

**Table 6:** Summary of antibiotics used for treatment of CDI.

Treatment	Toxin-Producing CD Strains (Presumptive 027/NAP1/BI)		*p*-Value
	Positive R1 (n = 43)	Negative R0 (n = 7)	
Vancomycin, n (%)	24 (55.8%)	1 (14.3%)	**0.049**
Metronidazole, n (%)	11 (25.5%)	5 (71.4%)	**0.027**
Vancomycin + Metronidazole, n (%)	8 (18.6%)	1 (14.3%)	0.630
Fecal microbiota transplantation, n (%)	2 (4.7%)	0 (0.0%)	0.737

**Table 7:** Outcome analysis of CDI.

Evolution	Toxin-Producing CD Strains (Presumptive 027/NAP1/BI)		*p-*Value
	Positive R1 (n = 43)	Negative R0 (n = 7)	
Recurrence rates, n (%)	14 (32.56%)	0 (0.0%)	0.025
Favorable evolution, n (%)	37 (86.0%)	6 (85.7%)	0.981
Mortality, n (%)	6 (14.0%)	1 (14.3%)	0.981

**Table 8:** Logistic regression models: predictors of mortality in group R1.

Logistic Regression Models Deceased, Presumptive 027/NAP1/BI Positive Assessed Variables	Odds Ratio (OR)	95% CI	*p*-Value
Calprotectin > 200	1.299	0.076–3.979	0.046
Calprotectin > 200 Toxin B (PCR)	2.364 2.159	0.411–13.584 0.610–12.543	0.034 0.001
Calprotectin 200 Toxin B (PCR) Number of stools	1.521 1.611 0.823	0.646–5.541 0.642–6.286 0.680–0.996	0.019 0.012 0.046

Abbreviations: CI—confidence interval; PCR—polymerase chain reaction.

**Table 9:** AUC values for laboratory parameters predicting mortality.

Test Result Variables	Area	Std. Error	Asymptotic Sig.	Asymptotic 95% Confidence Interval
Glucose	0.870	0.058	0.002	0.758–0.983
CRP at admission	0.761	0.081	0.028	0.603–0.919
CRP at discharge	0.892	0.071	0.001	0.753–1.000
Creatinine	0.826	0.057	0.006	0.714–0.937

Abbreviations: AUC—area under the curve. Note: The test result variable(s): glucose, CRP at discharge, and creatinine with at least one tie between the positive actual state group and the negative actual state group. Statistics may be biased: a. under the nonparametric assumption and b. when the null hypothesis has a true area = 0.5.

**Table 10:** Multimarker model for predicting mortality rates in group R1.

Model	R	R^2^	Adjusted R^2^	Std. Error of the Estimate	*p* Value	Durbin-Watson
1	0.370 ^a^	0.137	0.039	0.344	0.245	2.223
2	0.201 ^b^	0.040	−0.001	0.040	0.381	2.457
3	0.499 ^c^	0.249	0.233	0.307	<0.001	2.413
4	0.509 ^d^	0.259	0.227	0.308	0.001	2.347
5	0.566 ^e^	0.321	0.226	0.308	0.008	2.213

^a^ Predictors: (constant), toxin-B-positive PCR, fibrinogen, ESR, presumptive 027/NAP1/BI, CRP at admission. ^b^ Predictors: (constant), calprotectin 200 and toxin B. ^c^ Predictors: (constant), glucose. ^d^ Predictors: (constant), glucose and CRP at admission. ^e^ Predictors: (constant), ALT, platelets, CRP at admission, creatinine, glucose, and urea. Dependent variable: deaths.

## Data Availability

Data are contained within the article.
